# Palmitoyltransferase DHHC7 mediates protein palmitoylation and is essential for sperm function through the modulation of [Ca^2+^]_i_ and ROS signaling

**DOI:** 10.3389/fcell.2026.1849655

**Published:** 2026-06-01

**Authors:** Haixia Zheng, Xiaohong Cheng, Wenjie Hao, Peibei Sun, Yamei Xue, Jincui Ye, Xiumei Jiang, Changzhao Jiang, Kun Li

**Affiliations:** 1 School of Pharmacy, Hangzhou Medical College, Hangzhou, Zhejiang, China; 2 School of Basic Medical Sciences and Forensic Medicine, Hangzhou Medical College, Hangzhou, Zhejiang, China; 3 Assisted Reproduction Unit, Department of Obstetrics and Gynecology, Sir Run Run Shaw Hospital, Zhejiang University School of Medicine, Hangzhou, Zhejiang, China

**Keywords:** sperm, capacitation, palmitoyltransferase DHHC7, hyperactivation, calcium, motility, protein palmitoylation, reactive oxygen species

## Abstract

**Background:**

Palmitoylacyltransferase DHHC7 (DHHC7) plays a critical role in various biological processes and diseases. However, its expression, localization, and regulatory mechanisms in sperm function remain largely unknown.

**Methods:**

Immunofluorescent staining was used to localize DHHC7 in sperm and examine its colocalization with the estrogen receptor (ER), progesterone receptor (PR), and caveolin-1 (CAV1). To explore DHHC7’s role in sperm function, sperm were treated with a specific DHHC7 antibody. Subsequently, sperm motility and motion parameters were analyzed using a computer-assisted sperm analyzer, and the acrosome reaction (an indicator of sperm capacitation) was evaluated using fluorescein isothiocyanate-labeled Pisum sativum agglutinin. To explore the possible regulatory mechanisms of DHHC7 on sperm function, protein palmitoylation was assessed using the acyl-biotin exchange method. Intracellular calcium levels and reactive oxygen species (ROS) production were monitored using fluorescent probes Fluo-4 acetoxymethyl ester (Fluo-4 AM) and 2′,7′-Dichlorodihydrofluorescein diacetate (DCFH-DA), respectively, and protein tyrosine phosphorylation levels and DHHC7 protein in sperm were analyzed using Western blotting.

**Results:**

We found that DHHC7 was localized in the neck, principal piece, midpiece, and end piece of sperm in both mice and humans. Treatment with DHHC7 antibodies significantly impaired sperm motility, progressive movement, hyperactivation, and the acrosome reaction under capacitated conditions. Furthermore, DHHC7 colocalized with the ER, PR, and CAV1 in mouse sperm. Additionally, DHHC7 antibodies reduced protein palmitoylation and tyrosine phosphorylation levels, and reduced intracellular calcium elevation and ROS generation during mouse sperm capacitation.

**Conclusion:**

This study identifies DHHC7 as a regulator of sperm motility and capacitation and suggests that its effects involve palmitoylation-dependent modulation of calcium signaling, tyrosine phosphorylation, and ROS signaling by the spatial association with ER, PR, and CAV1. These findings provide novel insights into the biological functions and regulatory mechanisms of DHHC7 in sperm, highlighting its potential significance in reproductive biology.

## Introduction

Palmitoylacyltransferase DHHC7 (DHHC7) plays a pivotal role in numerous physiological and pathological processes. DHHC7 is one of the 24 members of the DHHC protein family, characterized by the Asp-His-His-Cys (DHHC) domain, and serves as a key palmitoyl acyltransferase in protein palmitoylation, a post-translational modification (Ko and Dixon, 2018). Protein palmitoylation, mediated by DHHC family enzymes, involves the covalent attachment of the 16-carbon saturated fatty acid palmitate to cysteine residues of target proteins, thereby modulating their function, localization, and signaling pathways (Jiang et al., 2018). Palmitoylation enhances protein hydrophobicity, regulates activity and stability, and is integral to membrane fusion and intracellular trafficking (Chamberlain and Shipston, 2015), and influences protein-protein and protein-lipid interactions (Greaves and Chamberlain, 2007). Recent studies have demonstrated that DHHC7 regulates diverse physiological and pathological processes, including pyroptosis (Zhang N. et al., 2024), intestinal fat absorption (Zhang F. et al., 2024), neurodevelopment (Hernandez et al., 2023), synaptic plasticity (Kerkenberg et al., 2021), inflammation (Yu et al., 2024; Wei et al., 2024), multiple malignancies (Jiang et al., 2023; Chen et al., 2016), and cardiac hypertrophy (Baldwin et al., 2023).

Despite DHHC7’s well-established roles in various cellular processes, its specific mechanisms of action in regulating sperm function remain unclear. Given that sperm function depends heavily on membrane-associated signaling, DHHC7-mediated palmitoylation may be particularly relevant in sperm physiology. Sperm function encompasses motility, hyperactivation, capacitation, and acrosome reaction (Aitken, 2006). Sperm motility is acquired in the epididymis and is hyperactivated during capacitation (Freitas et al., 2017). Capacitation, a complex event that occurs in the female reproductive tract, prepares spermatozoa for binding and fusion with the oocyte and is a prerequisite for mammalian fertilization, accompanied by cholesterol loss and plasma membrane remodeling. The acrosome reaction, a post-capacitation event, is also necessary for fertilization (Visconti et al., 2011). Sperm function relies on intricate signaling pathways. Intracellular calcium ([Ca^2+^]_i_) is crucial for sperm function, and [Ca^2+^]_i_-activated processes must be initiated at an appropriate time (Publicover et al., 2007; Finkelstein et al., 2020). Protein tyrosine phosphorylation is associated with sperm motility (Freitas et al., 2017) and capacitation (Naz and Rajesh, 2004; Visconti et al., 2011). Moreover, reactive oxygen species (ROS) play a crucial role in sperm function, and maintaining normal ROS levels is essential for normal sperm physiology. Conversely, increased ROS generation and decreased antioxidant capacity lead to oxidative stress in sperm (Chen et al., 2013). Furthermore, the estrogen receptor (ER) (Cooke et al., 2017; Luconi et al., 2004), progesterone receptor (PR) (Luconi et al., 2004), and caveolin-1 (CAV1) (Baltiérrez-Hoyos et al., 2012) proteins previously reported to be palmitoylated by DHHC7 in somatic cells (Pedram et al., 2012; Tonn Eisinger et al., 2018) play crucial roles in sperm function. Moreover, our previous study reported that protein palmitoylation in sperm (Li et al., 2017) is involved in sperm motility through calcium signaling, protein tyrosine phosphorylation, and ROS (Xiong et al., 2025), which may be related to DHHC7 function.

Given the critical role of protein palmitoylation in cellular function and the limited understanding of DHHC7’s role in sperm, we aimed to investigate its expression, hypothesize its involvement in regulating motility and capacitation, and elucidate the underlying mechanisms. This study may provide novel insights into the intricate mechanism that governs sperm function and potential targets for male contraceptive development and for treating male infertility.

## Materials and methods

### Reagents and animals

Human tubular fluid (HTF, MR-070-D) and palmostatin B (PSB, 178501, an inhibitor that blocks protein depalmitoylation) were obtained from Merck Millipore (Billerica, MA, United States). Progesterone (P4) was sourced from ICN Biomedicals (Irvine, CA, United States). PSA-FITC was purchased from Sigma-Aldrich. Solarbio Life Sciences (Beijing, China) supplied DCFH-DA, and dimethyl sulfoxide (DMSO) was procured from Merck (Darmstadt, Germany). Protease and phosphatase inhibitor cocktails were obtained from Roche (Mannheim, Germany). Fluo-4 AM, SDS lysis buffer (P0013G), and bicinchoninic acid assay (BCA) kits were obtained from the Beyotime Institute of Biotechnology (Shanghai, China). anti-β-tubulin antibody (ab6046; Cambridge, United Kingdom) was ordered from Abcam. BMCC-biotin and N-ethylmaleimide (NEM) were supplied by Thermo Fisher Scientific Inc. (Burlington, NC, United States). A pre-stained protein marker and an enhanced chemiluminescence (ECL) kit were acquired from Thermo Fisher Scientific (Waltham, MA, United States).

All animal experiments were conducted in accordance with the institutional guidelines of the Animal Care and Use Committee of Hangzhou Medical College. Sexually mature male ICR mice (>9 weeks old) were obtained from the Experimental Animal Center of Zhejiang Province. Animals were euthanized following the American Veterinary Medical Association Guidelines for the Euthanasia of Animals (2020). The animals were maintained in a specific pathogen-free (SPF) facility and housed in plastic enclosures. The facility was climate-controlled, maintaining a temperature of 23 °C ± 2 °C and a relative humidity of 60% ± 10%. A 12-h light-dark cycle was implemented, following protocols outlined in a previous study (Yi et al., 2022). The reports on animals in this study were in accordance with the ARRIVE guidelines.

### Antibodies

The following antibodies were supplied by Abcam (Cambridge, United Kingdom): rabbit anti-β-tubulin (ab6046); HRP-conjugated goat anti-rabbit secondary antibody (ab6721); goat anti-rabbit IgG H&L (Alexa Fluor® 488, ab150081); rabbit anti-ER (ab32063); and anti-caveolin-1 (ab32577). The mouse anti-DHHC7 antibody (H00055625-B01P) was purchased from Abnova (Taiwan, China) and supplied in PBS without preservatives. Antibody treatments were applied to intact sperm suspensions under capacitating conditions without any chemical permeabilization. Alexa Fluor 555 conjugated goat anti-mouse IgG (H + L) and F (ab′)2 fragments (4409S) were purchased from Cell Signaling Technology (Danvers, MA, United States). Purified mouse IgG antibody (026502) and phosphotyrosine polyclonal antibody (615800) were purchased from Invitrogen (Carlsbad, CA, United States). The PR antibody (AF6106-50) was provided by Affinity Biosciences (OH, United States).

### Sperm preparation

Mouse sperm were collected following a previously established protocol (Li et al., 2017; Olli et al., 2018). Sexually mature male mice in each experiment were euthanized using carbon dioxide, and their caudal epididymides were minced in pre-warmed HTF at 37 °C for 10 min to release sperm. The sperm suspension was separated from the epididymal tissue by centrifugation at 600 *g* for 10 s or by gravity settling. The supernatant was centrifuged at 500 *g* for 15 min. Sperm pellets were adjusted to approximately 2 × 10^7^ cells/mL using HTF.

This human sperm study was reviewed and approved by the Medical Ethics Committee of Hangzhou Medical College. Semen samples were donated by healthy men who provided written informed consent, and semen parameters were within the normal range according to the WHO standard: motility > 50% (grades a + b), concentration > 15 × 10^6^ cells/mL, and normal sperm morphology > 15% (World Health Organization, 2010). Sperm were then separated from seminal plasma by centrifugation at 800 *g* for 15 min after the semen was liquefied, as previously described (Cheng et al., 2023). To study the role of DHHC7, mouse sperm aliquots were treated with purified mouse IgG (a negative control) or DHHC7 antibody (H00055625B01P) at concentrations of 0.2 μg/mL or 2 μg/mL to neutralize DHHC7 activity and incubated under 5% CO_2_ at 37 °C, as described in the experimental design.

### Indirect immunofluorescence staining and protein colocalization analysis

Indirect immunofluorescence staining was performed as previously described (Li et al., 2014). Sperm samples were washed twice with PBS and fixed in 4% paraformaldehyde for 15 min before being smeared onto silane-coated slides (S4651-72EA; Sigma-Aldrich, MO, United States). After air-drying, the sperm were permeabilized with 0.1% Triton X-100 in PBS for 10 min, and then further washed with PBS at room temperature. Sperm were blocked with 10% goat serum at room temperature for 1 h. Sperm were then incubated with rabbit anti-ER, anti-PR, and anti-CAV1 antibodies (1:250) and mouse anti-DHHC7 antibodies (1:250) at 4 °C overnight. Normal rabbit IgG served as the negative control. Following three washes in PBS, sperm were incubated with Alexa Fluor 555-conjugated anti-mouse IgG and Alexa Fluor 488-conjugated anti-rabbit IgG (1:400) at 37 °C for 1 h. Sperm were washed three times with PBS before the cell nuclei were stained with DAPI for 5 minutes. Sperm were observed under a fluorescence microscope (Eclipse 80i; Nikon, Tokyo, Japan). Colocalization was quantified using the Coloc 2 plugin in Fiji/ImageJ. Pearson’s correlation coefficient was used to evaluate signal correlation, whereas Manders’ coefficients were used to assess spatial overlap.

### Protein extraction and western blot analysis

Protein extraction and Western blotting were performed as previously described (Li et al., 2014; Yi et al., 2022). Sperm samples and HEK293T cells were resuspended in SDS lysis buffer containing Roche Complete Mini EDTA-free Protease Inhibitor Cocktail and PhosSTOP phosphatase inhibitors. The suspension was centrifuged at 14,000 *g* for 15 min, and the supernatant was collected. The protein concentrations were measured using a BCA kit. The protein extracts in the loading dye were heated at 100 °C for 5 min. Subsequently, the proteins were resolved on a 10% SDS-PAGE gel and transferred to PVDF membranes (Millipore Corporation, Bedford, MA, United States). The membranes were blocked with TBS containing 0.01% Tween-20 (TBST; vol/vol) and 3% bovine serum albumin for an hour before incubation with primary antibodies at 4 °C overnight. After washing with TBST, the membranes were incubated with horseradish peroxidase-conjugated secondary antibodies for 2 h at room temperature. Protein bands were detected using an Enhanced Chemiluminescence Kit (Pierce Biotechnology, Rockford, IL, United States) and imaged using an Amersham Imager system (AI600QC, GE, United States). The molecular weights of the target proteins were determined using pre-stained protein markers. Membranes were stripped and re-probed with β-tubulin antibodies as loading controls. The target proteins were quantified from band intensities and analyzed using ImageJ.

### Evaluation of sperm motility and hyperactivation

Sperm motility and hyperactivation were evaluated using a computer-assisted sperm analyzer (CASA; IVOS; Hamilton-Thorne Biosciences, Beverly, MA, United States), as previously described (Li et al., 2014). The following CASA parameters were set to detect mouse sperm: frame rate, 60 Hz; acquisition frame, 45; minimum contrast, 50; minimum cell size, 5 pixels; cell intensity, 90; path velocity, 10 μm/s; straightness threshold, 0; slow cell, average path velocity (VAP) less than 5.0 mm/s and straight-line velocity (VSL) of 0 mm/s; and temperature of 37 °C. An aliquot of 5 µL sperm was placed into the chambers at a depth of 20 µm and pre-warmed to 37 °C. The following parameters were evaluated for at least 200 motile sperm: percentage of motile, progressive, and hyperactivated sperm; VSL; VAP; beat-cross frequency (BCF); amplitude of lateral head displacement (ALH); curvilinear velocity (VCL); linearity (LIN = VSL/VCL × 100); and straightness (STR = VSL/VAP × 100). Hyperactivation was defined using the SORT function as follows: ALH ≥ 7.0 µm, VCL ≥ 150 μm/s, and LIN ≤ 50%.

### Assessment of acrosome reaction

After treatment with the DHHC7 antibody under capacitated conditions for 60 min, sperm were incubated with 15 μM P4 for 15 min to induce the acrosome reaction. PSA–FITC staining was performed as previously described (World Health Organization, 2010). Sperm were washed twice with PBS for 5 min each. Sperm were fixed using ethanol for 30 min, smeared on slides, and air-dried. Sperm were then stained with 25 mg/L PSA–FITC at 4 °C in the dark overnight and washed three times with PBS. Sperm were observed under a fluorescence microscope with oil immersion at 450–490 nm excitation. At least 200 sperm in each group were categorized as follows (World Health Organization, 2010): acrosome-reacted (AR), sperm with only a green band at the equatorial segment or no fluorescing stain in the acrosome region; acrosome-intact (AI), sperm in which more than half of the head was bright and uniformly green. The percentage of sperm that underwent an acrosome reaction was then calculated.

### Detection of protein palmitoylation

Protein palmitoylation was assessed using the acyl-biotinyl exchange (ABE) method, as previously described (Xiong et al., 2025). The washed sperm were resuspended in cell lysis buffer (P0013, BIB, Shanghai, China) containing 50 mM N-ethylmaleimide (NEM) and incubated. Pellets were collected by centrifugation at 13,000 *g* for 15 min at 4 °C, and the protein extracts were collected and precipitated with ice-cold acetone at −20 °C for 90 min. The samples were divided into two aliquots: one was incubated with 1.5 M hydroxylamine at 4 °C for 2 h, and the other was treated with 0.1 M Tris-HCl as a control. Both aliquots were again precipitated with pre-cooled acetone, and the pellets were mixed and incubated with 0.8 µM BMCC-biotin in Tris-HCl buffer for 60 min at 4 °C. After further precipitation with pre-cooled acetone, the pellets were solubilized in the lysis buffer. Subsequently, the proteins were resolved on a 10% SDS-PAGE gel, transferred to PVDF membranes (Millipore Corporation, Bedford, MA, United States), and blocked with TBS containing 0.01% Tween-20 (TBST; vol/vol) and 3% bovine serum albumin for an hour before incubation with streptavidin-conjugated horseradish peroxidase.

### Measurement of [Ca^2+^]_i_

The [Ca^2+^]_i_ levels were measured using a previously established method (Xiong et al., 2025). Before capacitation, sperm samples were loaded with Fluo-4 AM (5 μM), a Ca^2+^ fluorescent probe, and incubated in the dark at 37 °C for 30 min. After four washes with HTF to remove excess dye, the sperm were incubated for an additional 20 min. Sperm (10^6^ cells/mL) were then added to a 96-well plate and treated with IgG and DHHC7 antibodies. The fluorescence intensity correlating to [Ca^2+^]_i_ was recorded at an excitation wavelength of 488 nm and an emission wavelength of 516 nm at 37 °C using a plate reader. Data were collected every 3 min for 30 min. The initial fluorescence values served as a standard for normalizing the subsequent intensity readings.

### Measurement of ROS levels

ROS levels were monitored using the fluorescent probe DCFH-DA as previously described (Xiong et al., 2025). Sperm samples were incubated with 10 μM DCFH-DA at 37 °C for 30 min, followed by five washes with HTF to remove the unbound probe. The sperm were then incubated for an additional 20 min. The sperm suspension (10^6^ cells/mL) was placed in a 96-well plate and treated with IgG and DHHC7 antibodies. Fluorescence signals were recorded at excitation/emission wavelengths of 488/529 nm at 37 °C using a plate reader. The fluorescence intensity was directly proportional to ROS levels. Data were captured at 3-min intervals over 30 min. The initial fluorescence values were used to standardize subsequent measurements.

### Statistical analysis

Data are presented as mean ± standard error of the mean (SEM). Different experiments were performed with 3 or 4 independent biological replicates, each from 1 to 3 individuals. A *post hoc* power analysis was conducted using G*Power 3.1 to confirm that the sample sizes were adequate. Statistical analyses were conducted using SPSS software (Version 26.0, IBM, United States). A one-way ANOVA was used to assess differences between groups. The Least Significant Difference (LSD) test was applied if variance homogeneity was confirmed (P > 0.05); otherwise, Dunnett’s T3 test was used. A P-value < 0.05 was considered indicative of statistical significance (two-tailed).

## Results

### Expression and localization of DHHC7 protein in sperm

**FIGURE 1:**
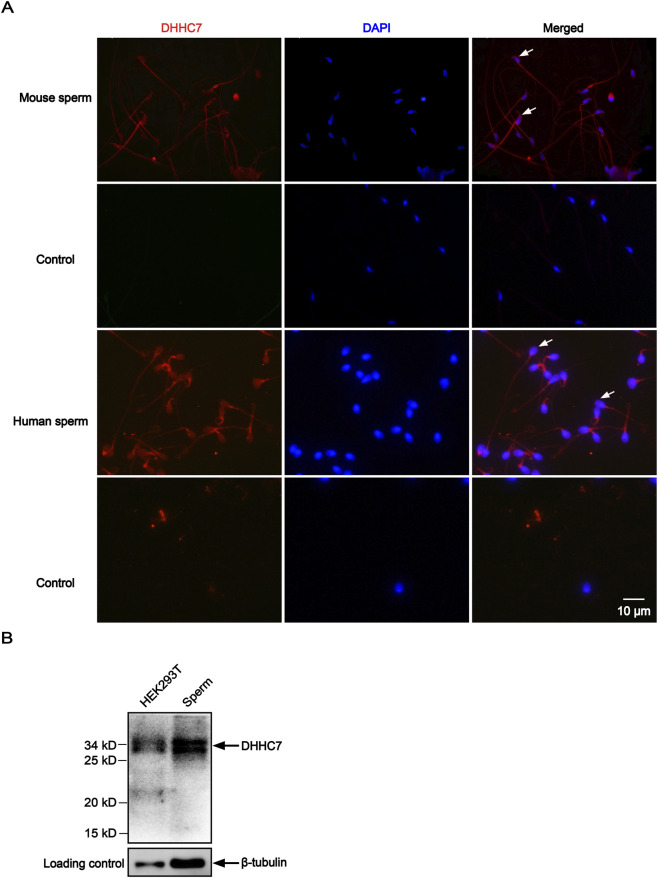
Localization and expression of DHHC7 in sperm. **(A)** DHHC7 is present in both mouse (top panel) and human (bottom panel) sperm. Sperm were stained by indirect immunofluorescence staining. DHHC7, with conjugated Alexa Fluor 555 in red, and nuclei, with 4′,6-diamidino-2-phenylindole (DAPI) in blue. A negative control below the corresponding DHHC7 staining using IgG instead of the primary antibody is shown. Arrows indicate acrosomes with weak red fluorescence. **(B)** DHHC7 in mouse sperm confirmed by Western blot analysis. Sperm (Sperm) lysates were immunoblotted with anti-DHHC7 antibody, with HEK293T (HEK293T) cells used as positive controls. An anti-β-tubulin antibody was used as a loading control. DHHC7, a specific band at approximately 35 kD, was observed. The loaded protein for the positive control was 5 μg/lane, based on the high abundance of DHHC7 in HEK293T cells in the preliminary experiments; the sperm sample was loaded at 30 μg/lane. These experiments were repeated three times using different samples. Scale bar, 10 µm.

To investigate DHHC7 expression in sperm, we used indirect immunofluorescence and Western blotting. Indirect immunofluorescence staining showed red fluorescence predominantly in the neck, midpiece, principal piece, and end piece of the sperm, indicating substantial localization of DHHC7 within these segments (Figure 1A, top panel). Simultaneously, the fluorescent signal in the acrosome of the head was weaker than that in other segments. Furthermore, DHHC7 in human sperm was located in segments similar to those in mouse sperm (Figure 1A, bottom panel). Western blot analysis revealed a single band at approximately 35 kD (Figure 1B) in the sperm sample, consistent with the predicted molecular weight of DHHC7, as validated by the positive control, lysates of HEK293T cells. Additionally, the single band supported the specificity of the DHHC7 antibody in sperm and its treatment. These findings confirm the expression and tail-biased localization of DHHC7 in mammalian sperm.

### Effect of DHHC7 antibody on sperm motility and hyperactivation

**FIGURE 2:**
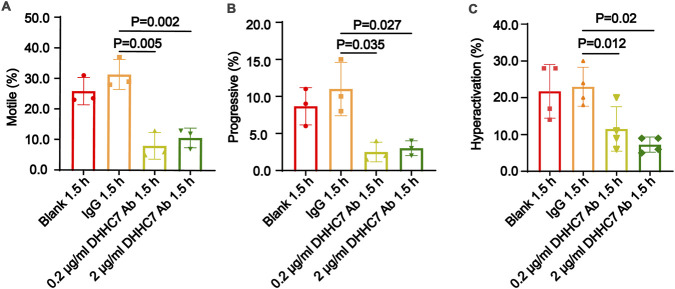
Effect of DHHC7 antibody on sperm motility and hyperactivation. Sperm motility and hyperactivation were evaluated as described in the Methods section. The results show the percentages of **(A)** motile, **(B)** progressive, and **(C)** hyperactivated sperm after treatment with the DHHC7 antibody. Data are presented as the mean ± SEM (n = 3 or 4 independent biological replicates from 1-3 individuals per replicate). A P-value < 0.05 signifies a statistically significant difference.

To explore the regulatory role of DHHC7 in sperm motility and hyperactivation, sperm parameters and hyperactivation were measured by CASA. The results showed a significant reduction in the percentage of motile (Figure 2A), progressive (Figure 2B), and hyperactivated sperm (Figure 2C) after mouse sperm were treated with 0.2 μg/mL or 2 μg/mL of DHHC7 antibody. No significant differences were observed in other motion parameters, including VCL, VSL, VAP, BCF, ALH, LIN, and STR (Supplementary Table 1). These results suggest that DHHC7 contributes to the regulation of sperm motility and hyperactivation.

### Effect of DHHC7 antibody on sperm capacitation

**FIGURE 3:**
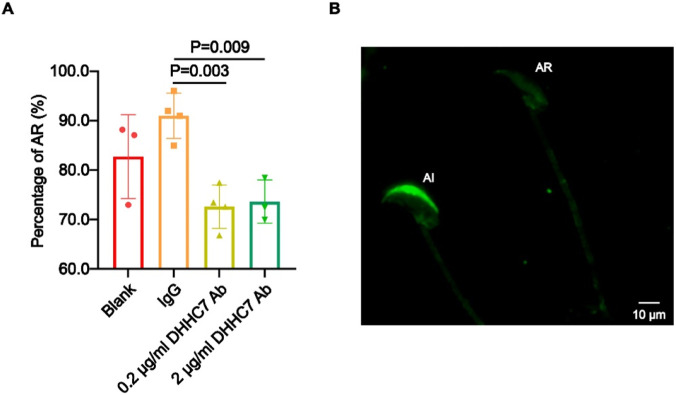
Effect of DHHC7 antibody on sperm capacitation. **(A)** Acrosomal reactions after sperm were treated with the DHHC7 antibody. Data are presented as the mean ± SEM (n = 3 or 4 independent biological replicates from 1-3 individuals per replicate). A P-value < 0.05 signifies a statistically significant difference. **(B)** The acrosome reaction was assessed using fluorescein isothiocyanate-labeled Pisum sativum agglutinin (PSA–FITC) staining. AR, sperm with reacted acrosomes; AI, sperm with intact acrosomes. Scale bar, 10 µm.

To examine the regulatory role of DHHC7 during sperm capacitation, we used *Pisum sativum* agglutinin labeled with fluorescein isothiocyanate (PSA–FITC) to assess the acrosomal reaction post-capacitation. The acrosomal reaction serves as an indicator of capacitation, occurring only in capacitated sperm. PSA–FITC staining distinguished the acrosomes in green that have not reacted (AI) from those that have reacted (AR) (Figure 3B). The results show that the percentage of the acrosome reaction in the group treated with DHHC7 antibody was significantly lower than that in both the IgG antibody control group and the blank control group (Figure 3A): the acrosome reaction rate decreased to 72.6% ± 2.2% in the 0.2 μg/mL DHHC7 antibody group vs. 91.0% ± 2.3% in the IgG control group (P = 0.003); similarly, treatment with 2 μg/mL of DHHC7 antibody reduced the percentage of acrosomal reaction to 73.6% ± 2.5%, from (91.0 ± 2.3)% in the control (P = 0.009). The differences observed between Blank and IgG control groups did not reach statistical significance (P > 0.05) and are consistent with normal biological variability between sperm preparations. These findings indicate that DHHC7 modulates sperm capacitation.

### Colocalization of DHHC7 with ER, PR, and CAV1 in sperm

**FIGURE 4:**
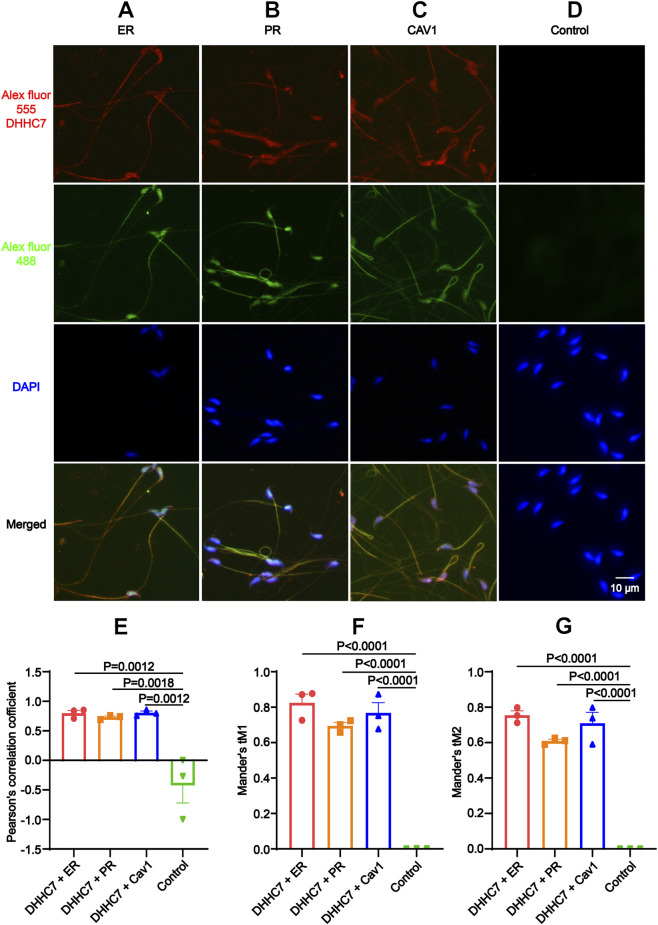
Colocalization of DHHC7 with ER, PR, and CAV1 in mouse sperm. DHHC7 was stained red using Alexa Fluor 555 conjugated to the anti-DHHC7 primary antibody, while **(A)** estrogen receptor (ER), **(B)** progesterone receptor (PR), and **(C)** caveolin-1 (CAV1) were stained green with Alexa Fluor 488, and nuclei were counterstained with 4′,6-diamidino-2-phenylindole (DAPI, blue). The results suggest the colocalization (yellow) of DHHC7 with ER, PR, and CAV1 in mouse sperm. **(D)** The rightmost panel shows a negative control using IgG instead of the primary antibody. The experiment was repeated four times using different samples. Colocalization quantification was performed using the Coloc 2 plugin in Fiji/ImageJ. **(E)** Pearson’s correlation coefficients, **(F)** Manders’ tM1, and **(G)** Manders’ tM2 were obtained by analyzing three different fields in each group. A P-value < 0.05 signifies a statistically significant difference. Scale bar, 10 µm.

To determine whether DHHC7 regulates sperm function via ER, PR, and CAV1, we examined colocalization by indirect immunofluorescence. The DHHC7 protein in red, stained with Alexa Fluor 555, was observed primarily in the neck and tail regions of the sperm, with minor expression in the post-acrosomal region of the head. Similarly, ER (Figure 4A), PR (Figure 4B), and CAV1 (Figure 4C), stained by Alexa Fluor 488, were expressed in the head and flagella, with a particularly strong signal in the midpiece and a weaker signal in the end-piece. Notably, the yellow signal after DHHC7 in red, which merged with ER, PR, and CAV1 in green, indicated that DHHC7 colocalized with ER, PR, or CAV1 in sperm. Coloc 2 analyses confirmed prominent colocalization of DHHC7 with ER, PR, and CAV1. In Figure 4E, Pearson’s correlation coefficients for DHHC7-ER, DHHC7-PR, and DHHC7-CAV1 were all above 0.5 and significantly higher than those in the control group (P = 0.0012, P = 0.0018, and P = 0.0012, respectively), reflecting substantial spatial overlap. The control group exhibited near-zero or negative correlation values, indicating negligible colocalization. Both Manders’ tM1 (Figure 4F) and tM2 (Figure 4G) in the DHHC7 groups were markedly elevated above 0.5 relative to control, with all differences highly significant (P < 0.0001). Collectively, these data demonstrate strong colocalization of DHHC7 with ER, PR, and CAV1, suggesting potential protein-protein spatial association and functional interactions among these molecules.

### Effect of DHHC7 antibody on levels of protein palmitoylation in sperm

**FIGURE 5:**
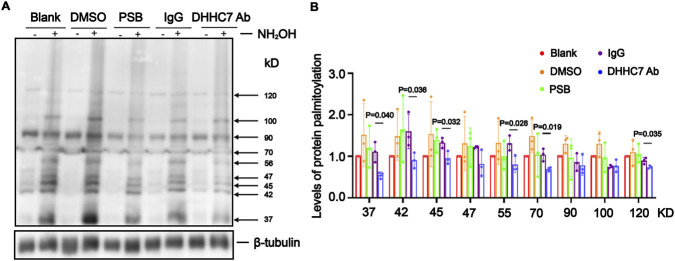
Effect of DHHC7 antibody on protein palmitoylation in sperm. **(A)** Protein palmitoylation levels in mouse sperm were assessed following sperm lysis and sodium dodecyl sulfate-polyacrylamide gel electrophoresis (SDS-PAGE) using the acyl-biotinyl exchange (ABE) method, and the loading controls of proteins were probed with an anti-β-tubulin antibody (ab6046; Abcam; Cambridge, United Kingdom) using Western blotting. All samples were treated for 90 min. Blank, untreated control; DMSO (dimethyl sulfoxide), vehicle control; PSB (palmostatin B, an inhibitor that blocks protein depalmitoylation, 50 μM), inhibitor control; IgG, 2 μg/mL IgG treatment, antibody control; DHHC7 Ab, 2 μg/mL DHHC7 antibody. **(B)** Quantitative analysis of protein palmitoylation levels. The gray intensities of the bands indicated by the arrows in **(A)** were quantified using ImageJ software. The gray intensity ratios of the bands were normalized to the loading control in the same lanes. Results are expressed as the mean ± SEM (n = 3 independent biological replicates from 1–3 individuals per replicate); *P < 0.05, considered statistically significant.

To further explore how DHHC7 influences sperm function through protein palmitoylation, protein palmitoylation levels in the sperm were examined. The results showed that DHHC7 antibody treatment altered palmitoylation levels of proteins in mouse sperm during capacitation (Figure 5A). Mouse sperm were treated with 2 μg/mL DHHC7 antibodies, along with the following controls: blank (untreated), DMSO, or 50 µM PSB (an inhibitor of protein depalmitoylation). Palmitoylation levels of proteins at approximately 37, 42, 45, 55, 70, and 120 kD in sperm treated with the DHHC7 antibody were diminished compared to those in the control IgG group (Figure 5B). These results suggest that DHHC7 affects sperm function through protein palmitoylation.

### Effect of the DHHC7 antibody on protein tyrosine phosphorylation in sperm

**FIGURE 6:**
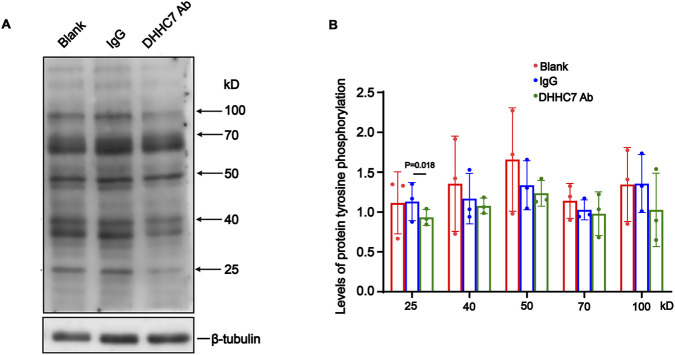
Effect of DHHC7 antibody on protein tyrosine phosphorylation in sperm. **(A)** Tyrosine phosphorylation levels in mouse sperm were evaluated using Western blotting. All samples were treated for 90 min. The tyrosine phosphorylation levels of the proteins were probed with a phosphotyrosine polyclonal antibody (615800; Invitrogen, Camarillo, CA, United States) following sperm lysis and sodium dodecyl sulfate-polyacrylamide gel electrophoresis (SDS-PAGE), and the loading control was anti-β-tubulin antibody (ab6046; Abcam, Cambridge, United Kingdom). Blank, untreated control; IgG, 2 μg/mL IgG treatment, antibody control; DHHC7 Ab, 2 μg/mL DHHC7 antibody. **(B)** Quantitative analysis of tyrosine phosphorylation. The gray intensities of the bands indicated by the arrows in **(A)** were quantified using the ImageJ software. The gray intensity ratios of the bands were normalized to the loading control in the same lanes. Results are expressed as the mean ± SEM (n = 3 independent biological replicates from 1-3 individuals per replicate); *P < 0.05, considered statistically significant.

However, whether DHHC7 controls sperm function through tyrosine phosphorylation remains unknown. The impact of the DHHC7 antibody on protein tyrosine phosphorylation levels was investigated. Western blot analysis showed that treatment with 2 μg/mL of the DHHC7 antibody reduced phosphotyrosine levels in multiple proteins (Figure 6A). The results showed that the tyrosine phosphorylation levels of proteins at approximately 25 kD were statistically significant, compared to IgG controls (Figure 6B). These results suggest that DHHC7 may regulate sperm function via the protein tyrosine phosphorylation signaling pathway.

### Effect of DHHC7 antibody on [Ca^2+^]_i_ levels in sperm

**FIGURE 7:**
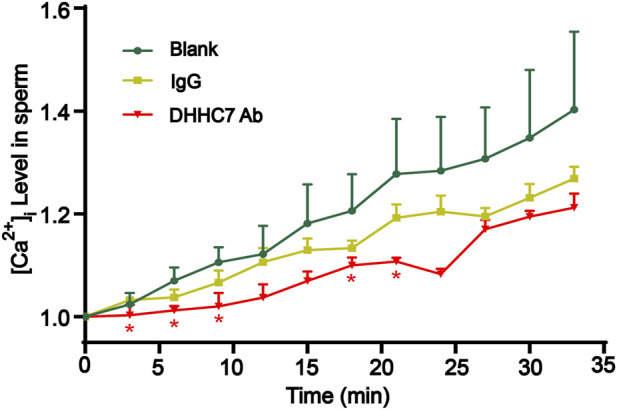
Effect of DHHC7 antibody on [Ca^2+^]_i_ levels in sperm. Mouse sperm were incubated with 5 μM Fluo-4, acetoxymethyl ester (Fluo-4 AM) in the dark at 37 °C for 30 min, followed by washing with HTF to remove unbound Fluo-4 AM. The fluorescence intensity was monitored at excitation/emission wavelengths of 488/516 nm. The intensity values were normalized to those of the blank group at 0 min. Blank, untreated control; IgG, 2 μg/mL IgG treatment, antibody control; DHHC7 Ab, 2 μg/mL DHHC7 antibody. Results are expressed as the mean ± SEM (n = 3 independent biological replicates from 1–3 individuals per replicate). *P < 0.05, indicating a significant difference between the treatment and control groups at the corresponding time points.

To study whether DHHC7 regulates sperm function through [Ca^2+^]_i_, the effect of the DHHC7 antibody on [Ca^2+^]_i_ levels in sperm was examined using Fluo-4 AM, a Ca^2+^ fluorescent probe (Figure 7). Mouse sperm loaded with Fluo-4 AM were incubated with 2 μg/mL DHHC7 antibody. The fluorescent signals were monitored for approximately 30 min. The data showed that the DHHC7 antibody reduced [Ca^2+^]_i_ levels (P < 0.05), suggesting that DHHC7 controls sperm function by modulating [Ca^2+^]_i_.

### Effect of DHHC7 antibody on ROS levels in sperm

**FIGURE 8:**
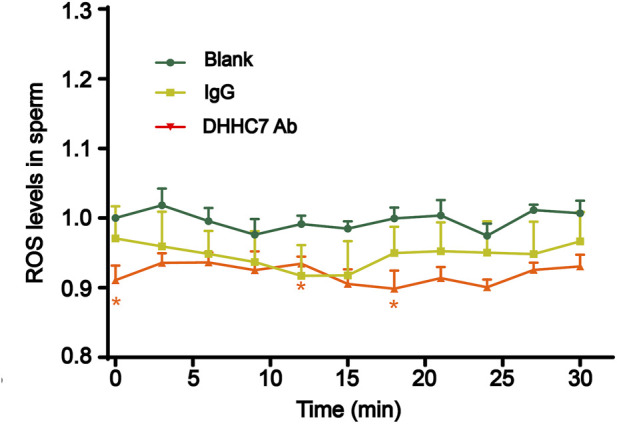
Effect of DHHC7 antibody on ROS levels in sperm. Mouse sperm were loaded with 10 μM Diacetyldichlorofluorescein (DCFH-DA) and washed with HTF to remove the unbound probe. The fluorescence intensity was monitored at excitation/emission wavelengths of 488/529 nm. The intensities were standardized to the 0 min fluorescence intensity. Blank, untreated control; IgG, 2 μg/mL IgG treatment, antibody control; DHHC7 Ab, 2 μg/mL DHHC7 antibody. Results are expressed as the mean ± SEM (n = 3 independent biological replicates from 1-3 individuals per replicate). *P < 0.05, indicating a significant difference between the treatment and control groups at the corresponding time points.

To investigate whether DHHC7 affects sperm function through ROS, the effect of the DHHC7 antibody on ROS levels in sperm was monitored with the fluorescent probe DCFH-DA (Figure 8). Mouse sperm loaded with DCFH-DA were incubated with 2 μg/mL DHHC7 antibody. The fluorescent signals were monitored for approximately 30 min. The data showed that the DHHC7 antibody reduced ROS levels (P < 0.05) compared to the IgG antibody control group, suggesting that DHHC7 regulates sperm function via modulating ROS production.

## Discussion

To the best of our knowledge, this is the first study to report the expression and localization of DHHC7 in mouse and human sperm and to investigate its role in regulating sperm function, expanding on its known biological roles. Our findings demonstrated that DHHC7 was predominantly located in the neck, principal, midpiece, and end piece of mouse sperm (Figure 1A), as confirmed by Western blotting (Figure 1B). In humans, DHHC7 expression is similar to that observed in mice. These localization patterns of DHHC7 suggest a pivotal role in specific sperm functions. Previous studies have reported that DHHC7 is localized in the Golgi apparatus in somatic cells (Jiang et al., 2018). However, our results revealed that DHHC7 is present in multiple regions, including the head and tail of the sperm, not in the Golgi apparatus. This finding suggests that DHHC7 may confer multiple functions in sperm. The localization of DHHC7 in the tail suggests it can regulate sperm motility, while its presence in the head suggests involvement in physiological processes such as capacitation. This finding expands the previous dogma that DHHC7 is expressed only in the Golgi apparatus, although the Golgi apparatus is also linked to acrosome formation. Therefore, it is speculated that DHHC7 is localized on the sperm plasma membrane, given the absence of the Golgi apparatus and endoplasmic reticulum in mature spermatozoa. This finding facilitates the functional exploration of DHHC7 not only in sperm but also in other somatic cells, as its plasma membrane distribution in sperm has not been previously documented. Collectively, these results indicate that DHHC7 plays diverse biological roles in sperm.

Our findings revealed that DHHC7 regulates sperm function, including motility and capacitation. The percentage of motile, progressive, and hyperactivated sperm was reduced following incubation with DHHC7 antibodies (Figure 2). These results suggest that DHHC7 plays a significant role in regulating sperm motility and capacitation (Figure 3). Notably, the functional effects observed at both 0.2 μg/mL and 2 μg/mL antibody concentrations were comparable, suggesting that the lower concentration may already achieve near-complete saturation of accessible DHHC7 epitopes, resulting in a plateau effect. This phenomenon may reflect a “plateau” or saturation effect, because it is highly likely that the accessible pool of DHHC7 on the cell surface in sperm is limited. Therefore, the lower concentration (0.2 μg/mL) may already have been sufficient to saturate these accessible targets, meaning a higher dose (2 μg/mL) could not elicit additional statistical inhibition. In addition, unlike chemical drugs that show a linear dose effect relationship, antigen and antibody binding follow the proportional rule and depend on their optimal ratio, not absolute dosage. The DHHC7 expression pattern in sperm supports this finding: DHHC7 localization in the sperm tail supports its role in motility, whereas its presence in the head suggests involvement in capacitation and the acrosome reaction.

Furthermore, the specific DHHC7 antibody could affect DHHC7’s catalytic activity by neutralizing antigen-antibody binding. In this study, sperm were not subjected to membrane permeabilization prior to DHHC7 antibody treatment. The biological effects of the DHHC7 antibody may be mediated through the following mechanisms. First, the anti-DHHC7 antibody may bind to an extracellular domain of DHHC7, thereby inducing conformational changes in the protein and modulating its catalytic activity. Mature sperm lack typical somatic-cell organelles, including the Golgi apparatus and endoplasmic reticulum, which supports the hypothesis that DHHC7 may localize to the sperm plasma membrane as a transmembrane protein. Previous evidence has demonstrated that DHHC7 is a multi-pass membrane protein. In the Golgi apparatus, DHHC7 contains cytoplasmic, luminal, and transmembrane domains; when localized to the plasma membrane, these topological regions of DHHC7 may correspond to intracellular, extracellular, and membrane-spanning regions, respectively. Second, the antibody may exert its effect by binding to epitope-containing peptides exposed in the extracellular region of DHHC7. The immunogen, used for antibody generation, corresponds to amino acids 1–208 of the full-length DHHC7 protein, according to the DHHC7 antibody datasheet. Moreover, extracellular segments of DHHC7 have been reported to contain epitope-like peptide sequences (Faridi et al., 2020), providing a structural basis for specific antibody binding. Third, the DHHC7 epitope recognized by the antibody, including regions associated with the catalytic domain, may become accessible only after capacitation-related membrane remodeling, the acrosome reaction, or transient membrane permeabilization. Capacitation, which was physiologically simulated in this experiment, induces extensive remodeling of the sperm plasma membrane, including cholesterol efflux, increased membrane fluidity, and lipid microdomain reorganization. These membrane changes may expose otherwise inaccessible DHHC7 epitopes, thereby facilitating antibody binding and subsequent functional modulation.

To date, no reports have indicated that DHHC7 regulates sperm function. DHHC19, another member of the DHHC protein family, has been reported to regulate sperm motility, acrosome reactions, and *in vitro* fertilization, although the exact molecular mechanisms remain unclear (Wang et al., 2021). This report also provides Supplementary Material, as the DHHC protein performs the same catalytic function in protein palmitoylation. Although the IgG control showed a slightly higher acrosome reaction rate than the blank control (Figure 3A), the difference was not statistically significant, supporting the use of IgG as an appropriate antibody control. This result may reflect the biological influence of supplemented IgG in the culture medium. As exogenous proteins similar to bovine serum albumin, IgG may slightly alter medium osmolarity and sperm membrane dynamics, thereby moderately affecting sperm biological signaling, further capacitation, and the acrosome reaction. When the IgG group serves as the control for the anti-DHHC7 antibody group, it eliminates interference from total protein concentration and non-specific steric effects in the medium, thereby verifying the specific effect of the anti-DHHC7 antibody variable region binding to DHHC7 protein. Consistent with the trend of the acrosome reaction rate, only minor numerical differences in [Ca^2+^]_i_ (Figure 7) and ROS level (Figure 8) are observed between the blank control and IgG control groups. Statistical analysis indicates no significant differences (P > 0.05) in the indicators detected between the two control groups. These findings further verify the reliability and consistency of results across independent experiments. Furthermore, the observation that a specific anti-DHHC7 antibody modulates sperm function and intracellular signaling provides evidence for the development of DHHC7-targeted antibody therapeutics.

DHHC7 appears to regulate sperm function by mediating protein palmitoylation. In the present study, treatment with a DHHC7 antibody altered the palmitoylation levels of sperm proteins across multiple molecular weight ranges (Figure 5), suggesting that DHHC7 inhibition affects a diverse set of substrate proteins. These results support a role for DHHC7 as an important palmitoyl acyltransferase in sperm. However, the specific DHHC enzymes responsible for protein palmitoylation in sperm have not yet been fully elucidated. Our previous study reported that protein palmitoylation is essential for sperm motility and is involved with calcium signaling, protein tyrosine phosphorylation, and ROS production (Xiong et al., 2025). Notably, several proteins reported to be palmitoylated by DHHC7 in other cell types are present in sperm and associated with sperm functions, including classic ER, PR, and androgen receptors (Pedram et al., 2012; Meitzen et al., 2013), cystic fibrosis transmembrane conductance regulator (McClure et al., 2014), Janus Kinase 1 (Hernandez et al., 2023), and heterotrimeric G protein alpha subunit (Tsutsumi et al., 2009). These findings suggest that DHHC7 modulates sperm physiology by palmitoylating multiple key regulatory proteins. Future studies are needed to identify DHHC7 substrates in sperm using proteomic and protein modification approaches.

DHHC7 may exhibit spatial association with ER, PR, and CAV1 in regulating sperm function. Previous studies have shown that DHHC7 is required for palmitoylation, membrane transport, and rapid signal transduction of endogenous ER and PR (Pedram et al., 2012), increases CAV1 palmitoylation (Tonn Eisinger et al., 2018), and that Erα interacts with CAV1 (Sosa et al., 2019). ER (Cooke et al., 2017; Luconi et al., 2004), PR (Luconi et al., 2004), and CAV1 (Baltiérrez-Hoyos et al., 2012) participate in the regulation of sperm function. The ER is associated with sperm motility, calcium mobilization (Saberwal et al., 2002), capacitation (Bosakova et al., 2018), and acrosome reactions (Gimeno-Martos et al., 2021), which interact with the PI3K/Akt pathway (Aquila et al., 2004) and are linked to tyrosine phosphorylation. PR is also involved in sperm motility and acrosome reactions (Gimeno-Martos et al., 2021; Sabeur et al., 1996; Li et al., 2014). CAV1 regulates sperm capacitation and reaction via interactions with CDC42 (Baltiérrez-Hoyos et al., 2012) and plasma membrane calcium ATPase 4 (Olli et al., 2018). Therefore, DHHC7 colocalized with ER, PR, and CAV1 in sperm (Figure 4). These results suggest that DHHC7 may be spatially associated with and palmitoylate ER, PR, and CAV1, although evidence that DHHC7 directly palmitoylates these proteins warrants further investigation. In summary, ER, PR, and CAV1 are involved in DHHC7’s regulation of sperm function.

Tyrosine phosphorylation is crucial for sperm motility and capacitation. Our results showed that the DHHC7 antibody reduced protein tyrosine phosphorylation levels (Figure 6), suggesting that DHHC7 likely influences sperm function through a protein tyrosine phosphorylation cascade. DHHC7 affects sperm tyrosine phosphorylation, possibly by palmitoylating kinases such as JAK (Hernandez et al., 2023) and STAT3, as reported in somatic cells (Jiang et al., 2023). These kinases have been reported to regulate tyrosine phosphorylation in sperm (Lachance and Leclerc, 2011). Furthermore, palmitoylation of STAT3 by DHHC7 is associated not only with tyrosine phosphorylation but also with ROS production in sperm (Lachance et al., 2016). This further corroborates the finding that DHHC7 affects ROS production in this study. In addition, there is crosstalk between calcium signaling and protein tyrosine phosphorylation in sperm. When DHHC7 affects protein tyrosine phosphorylation, calcium signaling is also affected.

[Ca^2+^]_i_ is essential for sperm motility and capacitation. Our study demonstrated that DHHC7 antibodies attenuated the increase in [Ca^2+^]_i_ levels in sperm (Figure 7), suggesting that DHHC7 modulates [Ca^2+^]_i_. ER, PR, and CAV1, colocalized with DHHC7, may regulate calcium signaling in sperm. Moreover, crosstalk with tyrosine phosphorylation affected by DHHC7 also induces [Ca^2+^]_i_ regulation. Additionally, the regulatory mechanism of calcium signals is complicated; there are diverse calcium channels, calcium pumps, and ion exchangers that affect calcium signaling and are likely DHHC7 substrates, such as CFTR (McClure et al., 2014), as in sperm reported by our lab (Li et al., 2010), which can be palmitoylated by DHHC7, documented in somatic cells. Together, it is reasonable that DHHC7 regulates sperm function via calcium signaling.

ROS plays a crucial role in the regulation of sperm function. Our observations indicated that exposure to DHHC7 antibodies transiently altered ROS levels during the initial 15 min, after which the differences gradually diminished (Figure 8). These findings suggest that DHHC7 dynamically influences ROS levels in sperm, implicating its role in ROS-mediated regulation of sperm function. The transient increase in ROS levels, followed by a return to baseline, suggests a biphasic response that may be crucial for processes such as sperm function, as moderate ROS levels are beneficial for physiological aspects such as motility, capacitation, and hyperactivation (Chianese and Pierantoni, 2021; Barati et al., 2020). Currently, there is limited evidence for a direct association between DHHC7 and ROS generation. However, evidence suggests that ROS generation is palmitoylation-dependent (Mohammed et al., 2013; Ge et al., 2022). Furthermore, protein palmitoylation has been shown to influence ROS levels. ROS can regulate [Ca^2+^]_i_ and protein tyrosine phosphorylation, which affects sperm capacitation (Serafini and O’Flaherty, 2022). Based on our results, it is reasonable to conclude that DHHC7 regulates sperm function by modulating ROS production.

Some limitations should be mentioned in this study. First, this study primarily used a DHHC7 antibody neutralization approach to explore the possible mechanism. The findings need further verification *in vivo* using a testis-specific DHHC7 knockout model in future research. Second, the palmitoylated substrate proteins of DHHC7 in sperm require further identification through systematic investigation in future work, although the total palmitoylated proteins were shown to be affected by DHHC7 in this study. Thirdly, the present study hypothesized that DHHC7 is localized to the plasma membrane rather than the Golgi apparatus, and that this localization pattern may also be across different cell types, which needs to be further validated in the future. Finally, the potential mechanism by which DHHC7 links palmitoylation to [Ca^2+^]_i_, ROS, and tyrosine phosphorylation in this study also needs to be further validated in subsequent studies.

## Conclusion

DHHC7 plays a crucial role in regulating sperm function, including motility and capacitation, and its regulatory mechanism may involve its colocalization with ER, PR, and CAV1, which triggers protein palmitoylation and subsequently activates downstream pathways through changes in [Ca^2+^]_i_, protein tyrosine phosphorylation, and ROS. This study highlights the significant role of DHHC7 in sperm physiological processes and provides insights into its regulatory mechanisms, providing a theoretical basis for developing male contraceptives and novel therapies for male infertility.

## Data Availability

The original contributions presented in the study are included in the article/Supplementary Material, further inquiries can be directed to the corresponding author.
