# Supplementary material for: Palmitoyltransferase DHHC7 mediates protein palmitoylation and is essential for sperm function through the modulation of [Ca2+]i and ROS signaling
Source: Front Cell Dev Biol. 2026 Jun 1;14:1849655. doi: 10.3389/fcell.2026.1849655 (PMC13265533; doi:10.3389/fcell.2026.1849655)
Supplement: Supplementary file 1 [file Table1.docx]

|  | **Blank 1.5 h**  **(n=3)** | **2 μg/mL lgG 1.5 h**  **(n=4)** | **0.2 μg/mL DHHC7 Ab 1.5 h**  **(n=3)** | **2 μg/mL DHHC7 Ab1.5 h**  **(n=4)** |
| --- | --- | --- | --- | --- |
| **VAP (μm/s)** | 35.5 ± 7.7 | 35.9 ± 6.0 | 33.7 ± 6.3 | 35.3 ± 7.0 |
| **VSL (μm/s)** | 23.4 ± 4.5 | 24.1 ± 3.3 | 17.5 ± 2.6 | 20.6 ± 4.2 |
| **VCL (μm/s)** | 70.4 ± 13.2 | 84.8 ± 10.3 | 74.4 ± 10.2 | 78.4 ± 12.6 |
| **ALH (μm)** | 4.5 ± 0.6 | 5.7 ± 0.6 | 5.0 ± 0.4 | 5.0 ± 0.4 |
| **BCF (Hz)** | 29.2 ± 2.0 | 29.1 ± 1.1 | 32.2 ± 0.7 | 32.2 ± 0.7 |
| **STR (%)** | 65.7 ± 6.1 | 61.0 ± 4.5 | 54.3 ± 4.4 | 54 ± 3.1 |
| **LIN (%)** | 38.0 ± 5.0 | 33.3 ± 2.9 | 28.3 ± 2.3 | 28.5 ± 1.4 |

**Table 1. Effect of DHHC7 antibody on motion parameters in mouse sperm**

VAP: average path velocity; VSL: straight-line velocity; VCL: curvilinear velocity; ALH: amplitude of lateral head displacement; BCF: beat cross frequency; STR: straightness (VSL/VAP×100); LIN: linearity (VSL/VCL×100); n, the number of independent replicates from different individuals. All data are presented as mean ± SEM.
